# Professor Simpson on Artificial Separation of the Placenta

**Published:** 1847-08

**Authors:** 


6. *Professor Simpson's Essay.*—III. *Artificial Separation of the Placenta.*—The arrestment of unavoidable flooding by total detachment of the placenta should, I believe, be our line of practice when the combination of circumstances is as follows, viz.: the hemorrhage is so great as to show the necessity of interference, and is not restrainable or restrained by milder measures (such as the evacuation of the liquor amnii); but at the same time, turning or any other mode of immediate and forcible delivery of the child, is especially hazardous or impracticable, in consequence of the undilated or undeveloped state of the os uteri, the contraction of the pelvic passage, &c. Or, again, the death, prematurity, or non-viability of the infant may not require us to adopt modes of delivery, for its sake, that are accompanied (as turning is) with much peril to the mother, provided we have a simpler and safer means, such as the detachment of the placenta, for at once commanding and restraining the hemorrhage, and guarding the life of the parent against the dangers of its continuance. Hence as I have elsewhere stated, I believe that the suppression of the flooding by the total detachment of the placenta will be found the proper line of practice in severe cases of unavoidable hemorrhage, complicatad with an os uteri so insufficiently dilated and undilatable as not to allow of version being performed with perfect safety to the mother;—therefore, in most primiparæ; in many cases in which placental presentations are (as very often happens) connected with premature labor and imperfect development of the cervix and os uteri; in labors supervening earlier than the seventh month; when the uterus is too contracted to allow of turning; when the pelvis or passages of the mother are organically contracted; when the child is dead; when it is premature and not viable; and where the mother is in such an extreme state of exhaustion as to be unable, without immediate peril of life, to be submitted to the shock and dangers of turning or forcible delivery of the infant. This enumeration is far from comprehending all the forms of placental presentations that are met with in practice; but it certainly includes a considerable proportion of the cases of this obstetric complication, and among all or almost all of the most dangerous and most difficult varieties of unavoidable hemorrhage. In adopting the practice, one error, which I would strongly protest against, has been committed in some instance. Besides completely detaching and extracting the placenta, the child has subsequently been extracted by direct operative interference. If the hemorrhage ceases, as it usually does, upon the placenta being completely separated, the expulsion of the child should be subsequently left to nature, unless it present preternaturally, or the labor afterwards show any kind of complication, which of itself would require operative interference under any other circumstances. Both to detach the placenta and extract the child would be hazarding a double instead of a single operation."—*Ibid.*

